# Design of Liver Functional Reserve Estimation Technique Based on Optical Densitometry

**DOI:** 10.3390/diagnostics10080599

**Published:** 2020-08-16

**Authors:** Ekaterina Savchenko, Ilya Kolokolnikov, Elena Velichko, Victor Osovskikh, Lyubov Kiseleva, Zhyldyz Musakulova

**Affiliations:** 1 Institute of Physics, Nanotechnology and Telecommunications, Peter the Great St. Petersburg Polytechnic University, 195251 St. Petersburg, Russia; kin_kolin@mail.ru (I.K.); velichko-spbstu@yandex.ru (E.V.); 2 Department of Anesthesiology and Resuscitation Russian Research Center for Radiology and Surgical Technologies, 197758 St. Petersburg, Russia; osoff@mail.ru (V.O.); luba_kiseleva@mail.ru (L.K.); 3 Computer Information Systems, International University of Kyrgyzstan, Bishkek 720007, Kyrgyzstan; mjyldyz@rambler.ru

**Keywords:** optical densitometry, liver diagnosis, indocyanine green, liver functional reserve, optical density, plasma disappearance rate

## Abstract

This work is aimed at creating a modified invasive technique for assessing the liver’s functional reserves. A study of the degree of hepatodepression is carried out by measuring the plasma elimination of indocyanine green using the method of optical densitometry. This paper presents test results for an aqueous solution and an albumin solution, as well as the results of measurements of plasma elimination of indocyanine green for patients with liver disease. Perfecting the proposed method will make an important scientific contribution to modern diagnostic medicine. Diagnosing the stages in the progression of the disease and its developing complications can make it possible to rapidly correct the patient’s treatment algorithm, achieving positive outcomes in medical practice.

## 1. Introduction

One of the pressing issues in modern hepatology is assessing the degree of hepatic depression [1,2,3,4,5]. As of 2019, 325 million people worldwide have been infected with viral hepatitis B or C, and 1.4 million people die from it every year, while the need for resection occurs in 80% of cases in patients with hepatitis C and B [2].

Liver disease can be divided into diffuse and focal. The underlying pathophysiological mechanisms of the progression of diffuse liver disease are inflammation, vascular insufficiency, or abnormal accumulation of substances. Chronic liver failure is most frequently caused by viral hepatitis and fatty degeneration of hepatocytes of alimentary origin. Fulminant liver failure is mainly caused by hepatitis viruses and xenobiotics (including drugs). Diffuse liver disease is traditionally treated by hepatologists. The progression of chronic liver failure naturally raises the question about a radical method of treatment that is liver transplantation. Unfortunately, the existing clinical criteria for prediction are not perfect, which means that patients are not placed on the waiting list for transplantation in a timely manner. In the case of focal lesions (primary tumor, metastases, parasitic cyst, and abscess), the surgical strategy consists of removing pathological lesions while ensuring sufficient function of remaining organ volume. Experimental data indicate that the mass of liver tissue sufficient to meet the needs of the body is about 1% of body weight. However, this refers to fully functional tissue. In case of combined focal and diffuse lesions, such estimates will be low. Another branch of medicine where functional assessment of the liver plays a crucial role is treatment of organ failure in patients in critical condition. It is known that the hepatic component is the most difficult to control.

The study of the functional status of the liver currently includes the parameters of synthesis (prothrombin, cholesterol, and albumin), cellular integrity (transaminase), detoxification (ammonia) excretion, and cholestasis (bilirubin, alkaline phosphatase, and gamma-glutamyl transpeptidase) in combination with various imaging techniques. These “static” tests of hepatic function have limitations, especially when it comes to prognosis of survival or liver function tests in patients in critical condition [6]. Currently, only dynamic tests based on studies of the clearance of exogenously introduced substances can give a global picture of organ function [7]. Some tests can be used repeatedly within a limited time frame, for example, allowing to determine the limit of resection more thoroughly in conditions of temporary vascular isolation of the organ.

Dozens of substances were proposed as dynamic markers (both natural participants of metabolic reactions, and xenobiotics), along with methods for their detection. Only a few have reached widespread clinical use. One of the markers that has become firmly rooted in the practice of hepatology is indocyanine green (ICG) [8]. It is a water-soluble non-toxic fluorescent dye used for diagnostics of the cardiovascular system since 1956 [9]. After indocyanine green is administered intravenously, it is distributed only to the intravascularly space, binding to plasma proteins, and is removed from the blood only by hepatocytes. ICG refers to exogenous markers with rapid hepatic elimination, respectively, the rate of excretion depends on the function of hepatocytes and the hepatic blood flow rate.

The gold standard for determining the rate of ICG elimination is the method of sequential samples. Blood samples taken at regular intervals after the introduction of ICG are centrifuged, and the plasma is subjected to photometry (spectrophotometry) at a wavelength of 805 nm [10,11]. The disadvantages of sequential sampling are the long times it takes, and the additional personnel required. Thanks to the unique physical characteristics of indocyanine green, determination of ICG elimination rate by pulse densitometry was first introduced in the late 1990s. While a large number of publications confirm a high correlation of invasive and non-invasive techniques, there are certain problems related to pulse densitometry [10,11,12,13,14]. In particular, impaired capillary blood flow [15,16], obesity, and tremor of the extremities prevent obtaining reliable data [14]. This problem is especially important) in patients in critical condition. If non-invasive measurements fail, not only are expensive drugs lost, but new measurements are inevitably delayed to ensure that the previous dose of the indicator has been eliminated. Unfortunately, the technique for non-invasive determination of absolute ICG concentration and the related opportunities for calculating the circulating blood volume have also not been developed any further.

To solve this problem, we propose to use a combination of invasive and non-invasive methods for determining the rate of dye elimination on a single platform, with the possibility of post-processing the data obtained by pulse densitometry. The goal of this work is to develop the first stage, namely, an invasive method for measuring the plasma elimination of indocyanine green for diagnosing hepatic function.

## 2. Experimental Setup of Optical Densitometry

**Figure 1:**
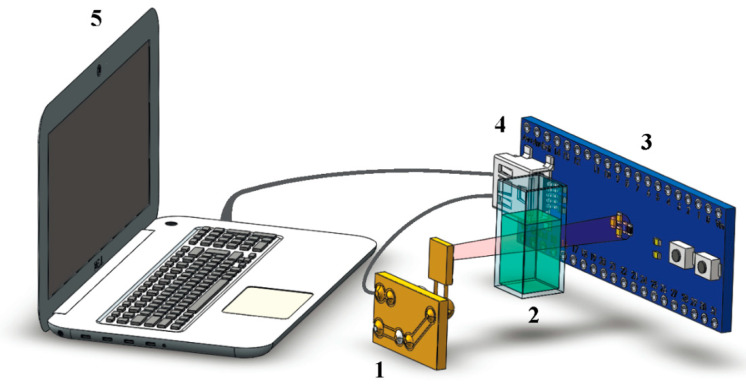
Experimental setup: (**1**) emitting diode, (**2**) sample cell, (**3**) photodetector, (**4**) microcontroller, and (**5**) computer.

An experimental setup that we developed (Figure 1) was used to conduct measurements of plasma elimination of indocyanine green for diagnostics of hepatic function.

Light emitted from diode (**1**) passes though the test solution. Part of the radiation is absorbed, and part of the radiation is backscattered [17,18]. The emitting diode has an emission spectrum narrower than the peak absorption spectrum of the dye in the near infrared region; therefore, a monochromatic filter in the circuit is not required. The prepared solution of known concentration of indocyanine green is placed in cuvette (**2**). Photodetector (**3**) is used to detect the transmitted radiation. The distance between the emitting diode and the photodetector is 100 mm. Measured at this distance, the intensity of light passing through the cuvette with water without dye is 100 µW. The cuvette with the solution is placed 50 mm from the emitting diode. Measurements are made every 0.4 s.

Microcontroller (**4**) controls the collection of data by the Serial Peripheral Interface bus. The emitting diode is powered by the MCU pins and has only two modes: on and off. Communication with the photodetector is performed through the I2C interface. Data from the microcontroller for further processing are displayed on user device (**5**), which is a personal computer or an android device via USB.

**Figure 2:**
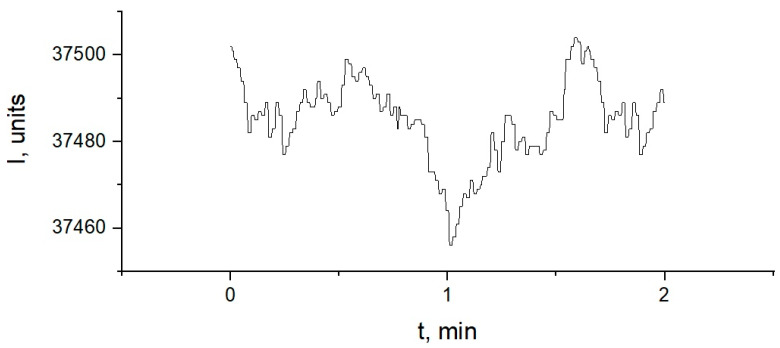
Example of time dependence of intensity of the light transmitted by 20% albumin solution without dye: I–the intensity of the light transmitted, t–recording time of the signal.

The signal fluctuations of the medium optical density occur during the measurements. Their deviation from the average value, as a rule, does not reach 0.5% [19,20]. Fluctuations in optical density can be conditioned by fluctuations of dye concentration, intensity measurement error is mostly determined by photodetector quantization error. Figure 2 presents an example of the received signal.

The developed experimental setup has several advantages compared with other photometric methods.

- Small dimension:
  The size of the experimental setup is 15 × 7 × 5 cm. The number of elements in this installation is minimized.
- Noise immunity:
  To improve the noise immunity, the Butterworth low pass filter and median filter were used.
- Cost-effectiveness:
  Optimized device design allows using cheaper optical elements. Emitting diode with 810 nm was used as a light source. (Which is cheaper than deuterium and tungsten sources)
- Simplicity of use:
  This experimental setup does not require additional training of users for carrying out experimental studies.
- Quick real-time presentation of the results:
  Processing of experimental studies takes up to several minutes.

## 3. Results and Discussion

This paper presents test measurements of the developed experimental setup and the proposed software. Solutions of the indocyanine green dye with water and with albumin were used as test samples.

**Figure 3:**
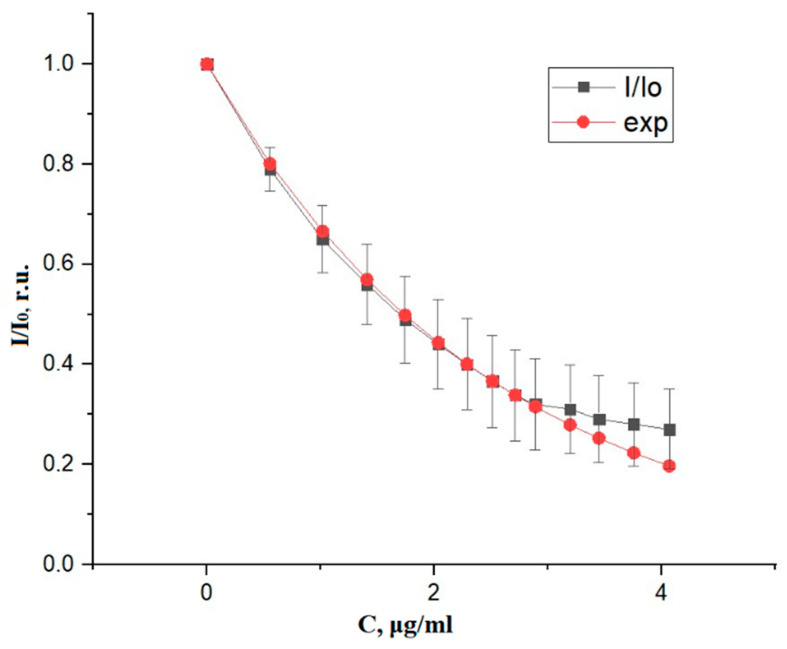
Ratio of intensity (I) of the light transmitted by aqueous solution of green indocyanine at low dye concentration (C) to intensity (I_0_) of the light transmitted by water, and its approximation by an exponential function (exp).

At the first stage, measurements were made for an aqueous solution of indocyanine green at a low dye concentration. These samples were prepared by adding small portions of the dye with a known concentration in distilled water. Figure 3 shows a graph of the obtained experimental results for measuring the concentration of an aqueous solution of green indocyanine at a low dye concentration and their approximation by the exponent. The measurement result is calculated by averaging the signal values. The error bars marked on the graph reflect the value of the random experimental error calculated by estimation of the mean value (10 measurements), and confidence interval with a confidence level of 95%.

Measurements at a low dye concentration are approximated by an exponent with a selected coefficient.

**Figure 4:**
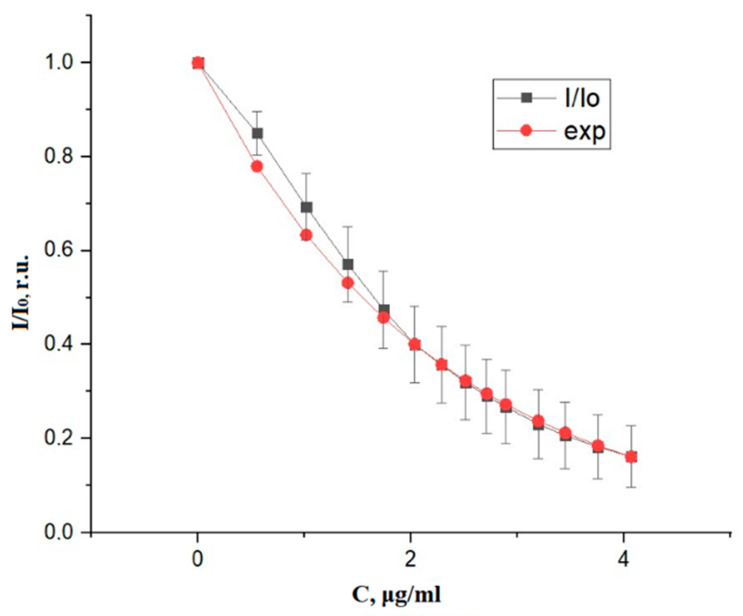
Ratio of the intensity of the light transmitted by dye albumin solution to intensity of light transmitted by solution of albumin and its approximation by an exponential function.

To test the operability of the developed experimental setup, we also performed experiments for a 20% albumin solution, gradually adding the dye to the test medium. The experimental results of measurements with low concentrations of indocyanine green in a 20% albumin solution and their exponential approximation are presented in Figure 4.

An experiment with a 20% albumin solution in the dye concentration range showed agreement with the theoretical data. However, in contrast to the experiment with water, it was not possible to choose an exponent that could approximate the beginning of the graph at a concentration near zero.

At the second stage of the measurements, we studied blood plasma samples from patients with liver pathology. Plasma samples were taken from 23 patients with liver pathology as part of routine ICG serial blood testing at the department of anesthesiology and intensive care of Russian Research Center of Radiology and Surgical Technologies. Plasma samples form a series each consisting of a set of measurements during the study of one patient. We have studied the samples taken before administering the dye, which are used to determine the optical density of the blood plasma itself. The intensity I_0_ of the light passing through the plasma without dye allows to calculate the relative concentration of the dye in subsequent samples:

(1) $$C_{rel}=-\ln\left(\frac{I}{I_{0}}\right).$$

**Figure 5:**
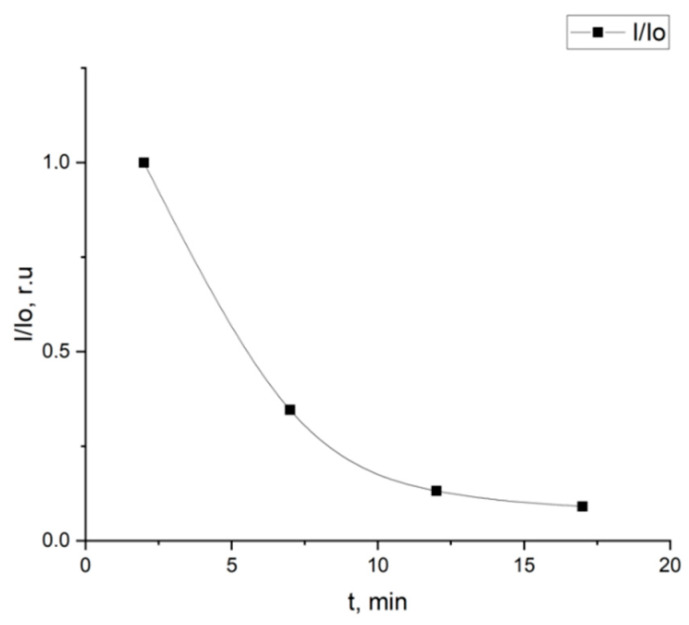
Ratio of intensity of the light transmitted by the dye blood plasma solution to the intensity of light transmitted by blood plasma solution from the time the plasma was taken from the patient.

In addition, plasma samples taken 2, 7, 12, and 17 min after dye administration were investigated. Figure 5 shows the results for a plasma sample from one of the patients with liver pathology.

**Figure 6:**
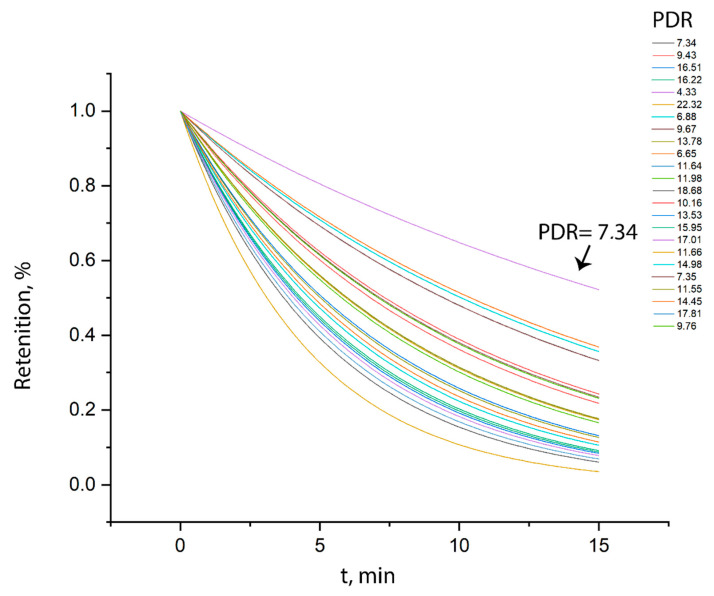
Plasma dye elimination curves corresponding to individual series.

Experiments with plasma allowed us to estimate the rate of dye elimination in the blood of patients. By calculating the relative concentration of the dye in these samples and comparing them, we can estimate the PDR (plasma disappearance rate) parameter by approximating a series of concentrations with an exponent with a selected coefficient for the argument. Measurements of the concentration ratio in the samples taken after 2 and 17 min determine the value of the PDR parameter, defined as the percentage by which the dye concentration decreases in 15 min. Figure 6 presents the results of experiments with plasma aimed at assessing the rate of elimination of the dye in the blood of patients.

The metabolic function of the liver of patients can be assessed based on these results. The faster the liver removes the dye from the circulatory system, the lower the ratio of the concentration in the subsequent sample to the concentration in the previous one after the same period. The liver of those patients whose curves are located lower purifies the blood better than the liver of those whose curves are higher.

**Figure 7:**
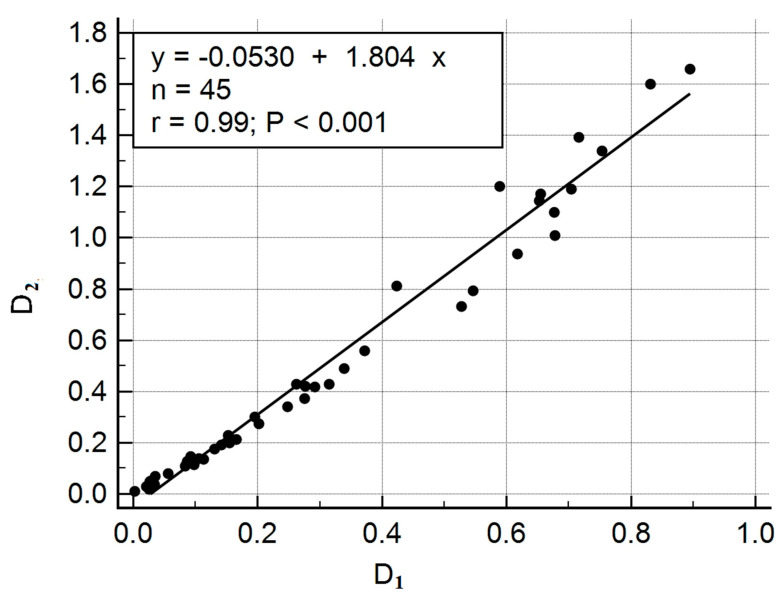
Comparison of the optical densities, D, obtained by two methods: D_2_–the optical densities of the samples measured using a spectrophotometer and D_1_–the optical densities of the samples measured using our experimental setup.

To compare the results obtained by evaluating the concentration of the dye in the plasma, parallel studies were carried out by the method of sequential sampling on a commercial DU^®^ 800 UV/Visible Spectrophotometer. Figure 7 shows the relationship between the optical densities of the samples measured using a spectrophotometer (D_2_) and the optical densities of the samples measured using our experimental setup (D_1_).

**Figure 8:**
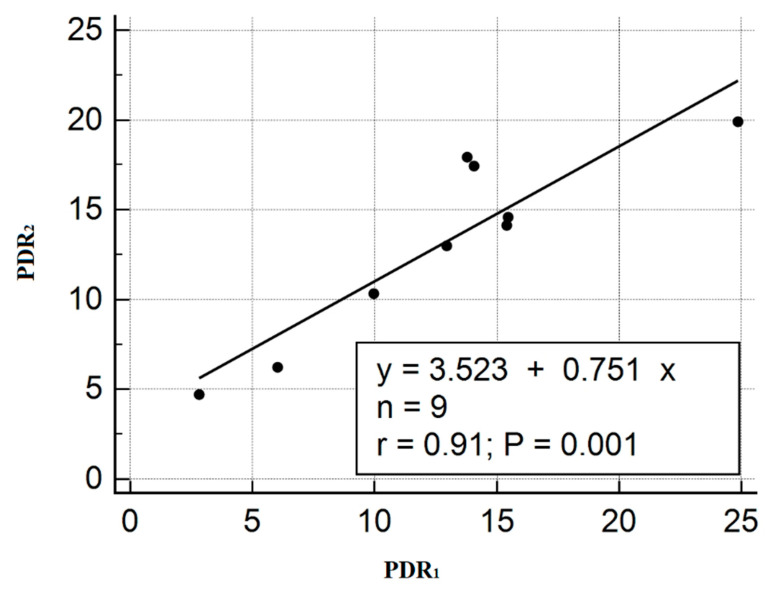
Comparison of plasma disappearance rate (PDR) parameters calculated from the measurements obtained with DU^®^ 800 UV/Visible Spectrophotometer at the Department of Anesthesiology and Intensive Care of Russian Research Center for Radiology and Surgical Technologies (PDR_2_), and with our experimental setup (PDR_1_).

PDR values were also calculated by two methods. The comparison of the results is shown in Figure 8.

Figure 7 and Figure 8 show that there is a correlation between the two measurement methods. The preliminary results obtained for assessing the concentration of indocyanine green in various liquids can be the basis for further studies [21]. This development can be included in the smart medical autonomous distributed system for diagnostics based on machine learning technology [22].

## 4. Conclusions

In this study, we developed an experimental setup, a program for collecting and displaying data, and an experimental technique for assessing the concentration of green indocyanine in various solutions. The experimental studies involved measurements with an aqueous dye, an albumin solution, and blood plasma correlated with the data from a commercial DU^®^ 800 UV/Visible spectrophotometer. The main (advantages) of a device are cost-effectiveness, simplicity of use and quick real-time presentation of the results. The developed system will make it possible to diagnose liver function and predict its recovery with higher accuracy compared with existing approaches.
